# Fatty Acid Profiling of Breast Milk at Different Gestational Ages

**DOI:** 10.3390/nu17162672

**Published:** 2025-08-19

**Authors:** Giuseppe De Bernardo, Giuseppina Leone, Federica Izzo, Marta Giovengo, Manuela Giovanna Basilicata, Fabio Centanni, Francesca Morlino, Emanuela Salviati, Maurizio Giordano, Serafina Perrone, Giuseppe Buonocore, Matteo Delli Carri, Giacomo Pepe, Pietro Campiglia

**Affiliations:** 1 Department of Woman and Child, Ospedale Buon Consiglio Fatebenefratelli, 80123 Naples, Italy; giuleo2787@gmail.com (G.L.); fedizz90@gmail.com (F.I.); 2 Department of Medicine and Surgery, “Scuola Medica Salernitana”, Section of Pediatrics, University of Salerno, 84084 Salerno, Italy; m.giovengo@studenti.unisa.it; 3 Division of Pediatrics, Department of Translational Medical Sciences, University of Naples Federico II, 80131 Naples, Italy; fabcent92@gmail.com (F.C.); francesca.morlino95@gmail.com (F.M.); 4 Department of Advanced Medical and Surgical Sciences, University of Campania “Luigi Vanvitelli”, 80138 Naples, Italy; mbasilicata@unisa.it; 5 Department of Pharmacy, University of Salerno, Via G. Paolo II, Fisciano, 84084 Salerno, Italy; esalviati@unisa.it (E.S.); mdellicarri@unisa.it (M.D.C.); gipepe@unisa.it (G.P.); pcampiglia@unisa.it (P.C.); 6 Department of Clinical Medicine and Surgery, Federico II University, 80131 Naples, Italy; mauri.giordano1990@gmail.com; 7 Neonatology Unit, Department of Medicine and Surgery, University of Parma, Pietro Barilla Children’s Hospital, 43121 Parma, Italy; serafina.perrone@unipr.it; 8 Department of Molecular and Developmental Medicine, University of Siena, 53100 Siena, Italy; giuseppe.buonocore@unisi.it; 9 PhD Program in Drug Discovery and Development, University of Salerno, Fisciano, 84084 Salerno, Italy

**Keywords:** fatty acids, newborn, breast milk, gestational age, mother, GC-MS

## Abstract

**Background/objectives:** This study aimed to characterize the fatty acid (FA) profile of breast milk (BM) at 7 days (T7) and 1 month postpartum (T30) using gas chromatography–mass spectrometry (GC-MS) and to evaluate associations between maternal diet during pregnancy and BM FA composition. **Methods**: A prospective observational cohort study was conducted from March 2022 to October 2023, involving mothers grouped by gestational age at delivery (32 weeks, 32–36.6 weeks, and >37 weeks). **Results:** BM lipid profiles were generally similar across gestational groups, with notable differences at T7 in saturated fatty acids (SFAs), myristic acid, monounsaturated fatty acids (MUFAs), erucic acid, nervonic acid, and some FA ratios. At T30, differences persisted in SFAs, MUFAs, myristic acid, and MUFA/SFA. At T7, red meat intake was positively correlated with stearic acid; white meat intake was negatively associated with multiple FAs (including **ω-3**) but positively with linoleic. Cheese correlated positively with caprylic acid; milk negatively with pentadecylic acid; and dried fruit positively with MUFA. At T30, fish consumption was prevalently positively related to DHA, EPA, and Omega-3, while red meat was positively associated with arachidic acid and margaric acid and negatively with di-homo-gamma linolenic acid. White meat showed a predominantly negative correlation with DHA, EPA and MUFA. Milk intake showed both positive (i.e., caproic acid) and multiple negative FA associations. Cheese was positively associated with caprylic acid, while dried fruit intake was positively linked to oleic acid and MUFA. **Conclusions:** Despite stable total lipid content, gestational age influenced specific FA profiles. These shifts may reflect adaptive responses to neonatal metabolic and neurodevelopmental needs. Understanding such mechanisms could guide tailored nutritional strategies, especially for preterm infants.

## 1. Introduction

Breastfeeding and human milk are the normative standards for infant nutrition [1]. Breast Milk (BM) is a dynamic and complex biological fluid and the optimal source of nutrition for newborns, as it provides species-specific nutrition that meets all the requirements for the child’s growth and maturation [2]. Moreover, BM is individualized because its nutrient composition adapts to meet the metabolic needs of the infant at various stages of life [3]. BM confers additional benefits in preterm infants, reducing prematurity-related morbidity and mortality [4]. Milk fat is primarily composed of triacylglycerols (TAGs), which account for more than 95% of its total lipid content. In addition to TAGs, minor components, such as di- and mono-acylglycerols, free fatty acids (FFAs), phospholipids, and cholesterol, are present [5]. The higher bioavailability and bioactivity of FFAs significantly contribute to the nutritional and developmental needs of neonates, particularly in supporting their immune function and growth. The composition of BM is influenced by various factors, including maternal diet, health status, and notably, the gestational age (GA) at which milk is produced [6]. Several studies have shown that the composition of BM from mothers of preterm infants is different to that of term infants, particularly in micronutrients, such as vitamins, and macronutrients, such as carbohydrates and proteins [7,8]. Lipids, which account for approximately 44% of the total energy intake in exclusively breastfed infants, are essential for numerous physiological processes, such as membrane synthesis, myelination, and the development of the infant’s immune system [9]. BM lipidic profile and the ratio of saturated fatty acids (SFAs) and monounsaturated fatty acids (MUFAs) are approximately constant; however, the concentrations of polyunsaturated fatty acids (PUFAs) can change [10]. Mature BM contain about 44% SFAs, about 39.9% MUFAs and about 20.6% PUFAs. Newborns should be fed with sufficient amounts of PUFAs, as they are related to infant visual activity, growth, and neurological development [11]. In the last trimester of pregnancy and in the first 18 months of the newborn’s life, DHA concentrates rapidly in the brain and regulates, together with AA, the genetic expression involved in neuronal growth and connections between nerve and glial cells [10,12,13,14]. A percentage of 50–60% of the dry weight of the brain is constituted of fatty acids, of which about 20–35% is constituted of **ω**-3 PUFA as DHA and EPA. DHA is the **ω**-3 fatty acid most represented in neuronal tissue and in grey matter, reaching a percentage of 40% [10,12,13,14]. However, EPA and DHA are slowly converted from the EFA precursor as ALA, with a rate conversion of 1% and 8%, respectively. EPA and DHA can also be assumed with a diet enriched with **ω**-3 fatty acids with cold water fish, such as salmon, tuna and herring, shellfish, human milk or supplements enriched with fish or microalgae oils. During pregnancy, it is recommended that mothers assume a supplementation of 100–200 mg/day DHA-EPA and consume two portions of fish per week [12,13,15]. BM lipidome analysis has a critical role in characterizing its composition and the differences at each stage of the newborn’s life [16]. In this work, the first aim was to test the DHA and lipidic profile of BM, expressed as grams per 100 g of total FFA, in milk samples collected at 7 days and 1 month postpartum from mothers who had given birth at different GAs. The secondary aim was to evaluate the correlation between the BM lipidic profile and maternal nutrition during pregnancy.

## 2. Materials and Methods

### 2.1. Study Design

A prospective observational cohort study was conducted at the Department of Woman and Child, Buon Consiglio Fatebenefratelli Hospital, Naples, Italy, from March 2022 and October 2023. This study is registered and publicly accessible at https://clinicaltrials.gov/study/NCT05989009, accessed on 18 July 2025. Inclusion criteria were mothers who have given birth at the hospital at any gestational age, written informed consent, and a balanced diet without dietary restrictions. Exclusion criteria were food intolerances, Celiac disease, vegan or vegetarian diet, diet excluding milk, diagnosis of metabolic diseases (e.g., diabetes, hypercholesterolemia, gout) and withdrawal of informed consent. The recruited mothers were divided into three groups based on the GA at birth:

Group < 32 wks (A): women who delivered before 32 weeks of GA;
Group 32–36.6 wks (B): women who delivered between 32 weeks and 36 + 6 weeks of GA’
Group > 37 wks (C): women who delivered after 37 weeks of GA.

A 10 mL milk sample was collected from each mother using a standard breast pump available in the department. The mothers were informed about the milk collection procedure by a nurse on the ward, which occurred at 12:00 and at the end of the lactation (hindmilk), two hours after the previous feeding. The milk samples were collected at 7 days, (transitional milk, T7) and 1 month postpartum (mature milk, T30) and transferred into test tubes, without the need for any type of preservative measures and stored at −20 °C until analysis. The milk samples were sent via courier to the centre responsible for the analytical study (Department of Pharmacy, University of Salerno). Clinical and nutritional data were collected from each mother by a health professional with a case report form (CRF) (Table S1) to investigate key conditions known in the literature for their ability to influence the lipid content of BM [17]. The CRF was divided into the following 4 sections:

Section 1: Personal Information

Basic maternal details were collected, such as name, age at delivery, height, pre-pregnancy weight, pregnancy weight gain, number of pregnancies, and type of infant feeding in previous pregnancies.

Section 2: Dietary Habits

The average weekly intake of various food groups during pregnancy was recorded, including fish, meats, dairy, eggs, fruits, and vegetables, along with details such as types of food consumed.

Section 3: Supplementation

Information was gathered about the use of dietary supplements during pregnancy based on DHA and EPA.

Section 4: Maternal Health

Maternal health was assessed by the presence of any medical conditions during pregnancy (e.g., gestational diabetes, hypertension).

This dietary recall instrument has not been validated or matched with any biomarker of nutrient intake.

### 2.2. Sample Preparation

Frozen breast milk samples were thawed to room temperature and vortexed for 1 min. Milk fat was extracted following the method described by Chen et al. [18]. Briefly, an aliquot of the milk sample was mixed with ammonium hydroxide solution, ethanol, and pyrogallic acid, and then hydrolysed at 70 °C for 20 min. To extract milk fat, a solution of n-hexane/isopropanol (3:2, *v*/*v*) was added to the hydrolysate, followed by vigorous agitation and centrifugation. The n-hexane phase was then collected and mixed with 2% methanolic sodium hydroxide, followed by heating and agitation at 50 °C for 20 min to perform base-catalysed methylation. After cooling, 100 μL of hexane and 100 μL of ultrapure water were added to enhance phase separation. Following centrifugation (10 min at 4 °C, 14,000 rpm), the upper n-hexane layer was collected, spiked with biphenyl in hexane (internal standard, IS), and analysed by GC–MS.

### 2.3. Gas Chromatography–Mass Spectrometry Analysis

Fatty Acid Methyl Esters (FAMEs) were analysed using a gas chromatography–mass spectrometry system (8890 GC coupled with a 5977B Mass Selective Detector, Agilent Technologies, Milan, Italy). Chromatographic separation was performed on a Zebron ZB-FAME capillary column (30 m × 0.25 mm × 0.20 μm, Phenomenex, Bologna, Italy). Electron ionization was carried out at 70 eV. The ion source and quadrupole temperatures were set at 230 °C and 150 °C, respectively. The injector inlet and MSD transfer line were maintained at 260 °C. The carrier gas was helium at a constant flow rate of 1.1 mL/min. The oven temperature program was as follows: initial temperature of 60 °C (held for 1 min), ramped at 9 °C/min to 150 °C (held for 1 min), followed by a ramp of 2 °C/min to 195 °C (held for 1 min). The total runtime was 36 min, followed by a post-run hold at 230 °C for 1 min and a 1 min equilibration period prior to the next injection. A solvent delay of 4 min was applied. FAMEs were detected in Selected Ion Monitoring (SIM) mode, as detailed in Table S2. Data acquisition and processing were performed using MassHunter Workstation Quantitative Analysis software (Version B.10.2; Agilent Technologies, Stockport, UK).

### 2.4. Calibration Curves and GC-MS Method Validation

The Supelco 37-Component FAME Mix (Bellefonte, PA, USA) was used as a stock solution. Aliquots were diluted to prepare a point calibration curve spiked with IS. Linearity was evaluated using the coefficient of determination (R^2^) calculated from the ratio of FAME to IS concentrations versus the corresponding peak area ratios. Fatty acid concentrations were determined by applying stoichiometric conversion factors and expressed as grams per 100 g of total FFA (*w*/*w*%). Method validation included the assessment of limits of detection (LOD), limits of quantification (LOQ), precision, and accuracy. LOD and LOQ were calculated using the standard deviation (SD) of the response and the slope of the calibration curve, as 3.3 × SD/slope and 10 × SD/slope, respectively. Precision was assessed in terms of intra-day and inter-day repeatability, expressed as relative standard deviation (%RSD). Accuracy was calculated as the percentage relative error (Er%). The method demonstrated acceptable linearity, precision, and accuracy (Table S3).

### 2.5. Calculation of Lipid Quality Indices Formulas

Lipid quality indices were calculated based on equations described by Chen J et al. [19] and Purkiewicz A et al. [20,21] as follows:

1. Index of atherogenicity (IA)= [C12:0 + (4 × C14:0) + C16:0]/∑unsaturated FA(UFA).
2. Index of thrombogenicity (IT) = (C14:0 + C16:0 + C18:0)/[(0.5 × ∑MUFA) + (0.5 × ∑n-6 PUFA) + (3 × ∑n-3 PUFA) + (n-3/n-6)].
3. Hypocholesterolemic/hypercholesterolemic ratio (HH) = (cis-C18:1 + ∑PUFA)/(C12:0 + C14:0 + C16:0).
4. Health-promoting index (HPI) = ∑UFA/[C12:0 + (4 × C14:0) + C16:0].
5. Unsaturation index (UI) = 1 × (% monoenoics) + 2 × (% dienoics) + 3 × (% trienoics) + 4 × (% tetraenoics) + 5 × (% pentaenoics) + 6 × (% hexaenoics).
6. Fish lipid quality/flesh Lipid quality (FLQ) = 100 × (C22:6 n-3 + C20:5 n-3)/SFA.
7. Linoleic acid/-linolenic acid ratio (LA/ALA) = C18:2 n-6/C18:3 n-3.
8. Trans fatty acid (TFA) = ∑TFA.
9. Index of Desirable Fatty Acids (DFA) = UFA + C18:0.
10. Index of Hypercholesterolemic Fatty Acids (OFA)= C12:0 + C14:0 + C16:0.
11. PUFA/SFA ratio = ∑PUFA/∑SFA.
12. PUFA/MUFA ratio = ∑PUFA/∑MUFA.
13. MUFA/SFA ratio = ∑MUFA/∑SFA.
14. ω-6/ω-3 ratio = ∑n-6 PUFA/∑n-3 PUFA.

### 2.6. Statistical Analysis

The sample size was calculated using G*Power 3.1.9.2 for Windows [22]. Due to the lack of available data, the sample size was estimated based on Cohen’s empirical rules, assuming a small effect size for DHA concentration across the three groups. According to Cohen (1969), conventional effect sizes are defined as small f = 0.14, medium f = 0.25, and large f = 0.40 [23]. Assuming an effect size of 0.25, α = 0.05, β = 0.05, number of groups = 3, number of measurements = 2, correlation between repeated measures = 0.3, and non-sphericity correction = 1, the required sample size was 90. This was the minimum number needed. Considering potential dropouts and estimating an overall loss of 18%, a total of 106 participants were recruited. The dataset of relative FAME concentrations, obtained via GC–MS and normalized to an internal standard, was assembled for multivariate statistical analysis. All computations were performed using MetaboAnalyst (v6.0; Xia Lab, McGill University, Montreal, QC, Canada), employing the platform’s built-in data processing pipeline. A comprehensive preprocessing procedure was applied to minimize the influence of technical variation and to prepare the data for subsequent chemometric analyses. The dataset was autoscaled by mean centring each variable and dividing by its standard deviation, ensuring all variables contributed equally to the multivariate models. Principal Component Analysis (PCA) was conducted as an unsupervised exploratory approach to identify patterns of variation and sample clustering based on FAME composition, while reducing dimensionality and experimental noise. The analysis was performed separately for each time point (T7 and T30), considering the three gestational age groups. The univariate statistical analysis was performed by a statistician aware of the study’s purpose using SPSS version 25.0 for Windows (IBM, Armonk, NY, USA). Normal distribution of the data was assessed using the Shapiro–Wilk test. Continuous and nominal data were reported as median and interquartile range or percentages, respectively. Non-parametric data were analysed using the Kruskal–Wallis test with Bonferroni post hoc corrections. Pearson’s correlation was used to determine relationships between continuous data. The heatmaps were generated in RStudio (version 2025.5.0.496) using the pheatmap package. Data with *p* < 0.05 were considered statistically significant.

## 3. Results

**Figure 1:**
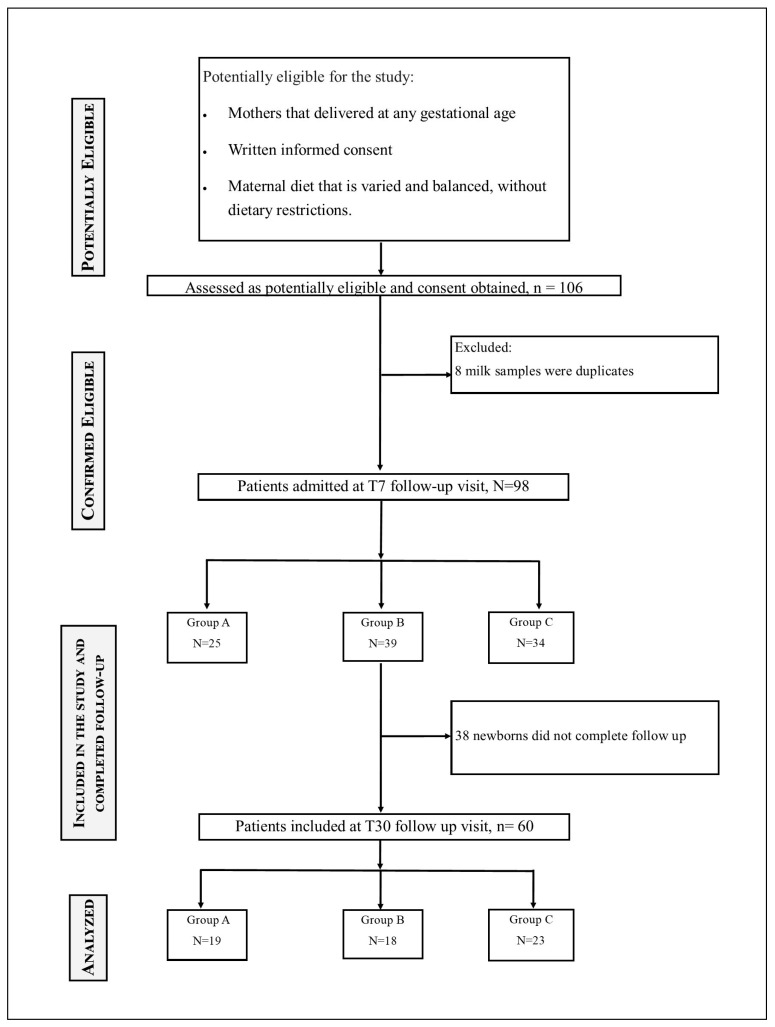
Flow chart diagram showing the enrolment and follow-up of the mothers.

**Figure 2:**
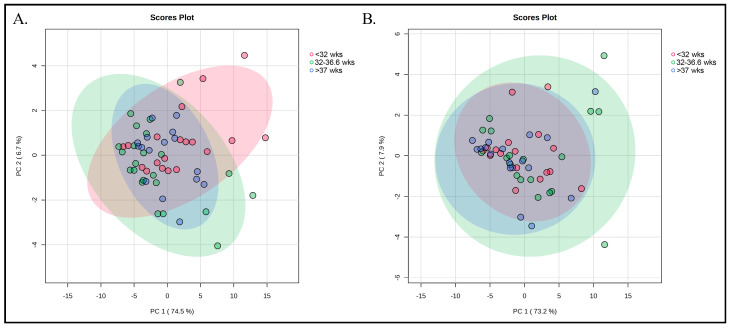
PCA score plots of relative FAME concentrations obtained via GC–MS and normalized to an internal standard. Data were autoscaled prior to analysis. (**A**) PCA for time point T7. (**B**) PCA for time point T30. Samples are coloured according to gestational age group: <32 wks group (red), 32–36 wks group (green), and >37 wks group (blue). Ellipses represent the 95% confidence regions for each group. PC1 accounted for 78.3% and 74.8% of the variance for T7 and T30, respectively, while PC2 explained 6.0% and 10.2%.

A total of 98 and 60 samples were collected at T7 and T30, respectively, as reported in Figure 1, and were analysed. The baseline characteristics of the mother enrolled in the study, such as age, body mass index, number of pregnancies, diet and DHA/EPA supplement intake were not statistically significant (Table 1). PCA was carried out separately for T7 and T30 to explore potential clustering of samples according to gestational age group. The resulting score plots (Figure 2) show that, in both time points, the three groups largely overlap between the first two principal components. For T7 (Figure 2A), PC1 and PC2 explained 78.3% and 6.0% of the total variance, respectively, whereas for T30 (Figure 2B), the corresponding values were 74.8% and 10.2%. No distinct separation patterns emerged, indicating that the variability in relative FAME profiles within each gestational age group was comparable to that observed between groups. The relatively small sample size and the high degree of homogeneity among the groups may have contributed to this lack of separation. Furthermore, the similarity in metabolic profiles could plausibly be related to comparable dietary habits and supplement intake among participants, which may have limited detectable differences in FAME composition among groups.

**Figure 3:**
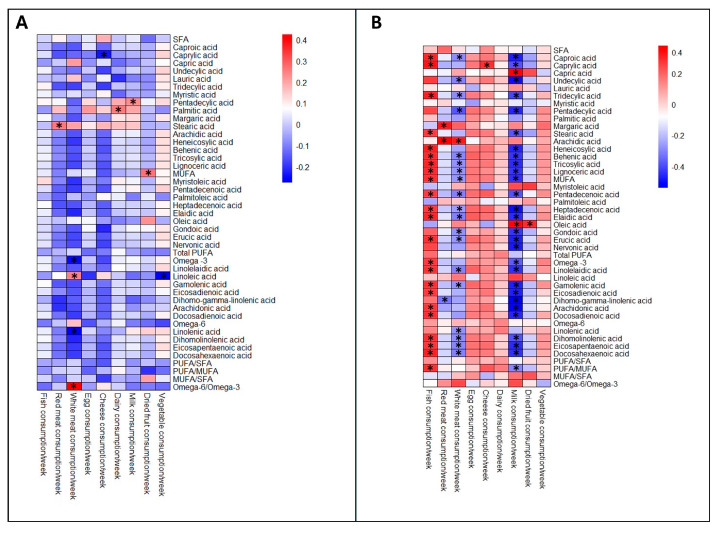
Heat map plot representing the correlation matrix between nutritional habits and fatty acids in breast milk. The intensity of the colour represents the degree of correlation. Positive correlation values are higher the more the colour tends toward red, while negative correlation values are lower the more the colour tends toward blue, as shown in the coloured box on the right. (**A**) T7 and (**B**) T30. * *p* < 0.05.

At T7 and T30, fatty acid concentrations were similar among groups (Table 2). Gestational age was correlated with myristic acid (r = −0.282; *p* = 0.007), SFA (r = −0.238; *p* = 0.023), and MUFA/SFA ratio at T7 (r = 0.269; *p* = 0.01), and with lauric acid (r = −0.289; *p* = 0.025), myristic acid (r = −0.341; *p* = 0.008), oleic acid (r = 0.338; *p* = 0.008), SFA (r = −0.373; *p* = 0.003), total MUFA (r = 0.391; *p* = 0.002), MUFA/SFA ratio (r = 0.360; *p* = 0.005) at T30. The analysis of the mother’s diet showed that weekly consumption of fish, red meat, white meat, cheese and dairy, milk, dried fruit and vegetables correlates with the lipid profile of BM (Figure 3, Tables S4 and S5). Lipid quality indices were also evaluated to enable a more comprehensive comparison of BM composition across different GAs. At time point T7, BM < 32 wks showed significantly higher AI, TI and OFA values and lower HH, UI, HPI, PUFA/SFA, MUFA/SFA and DFAs compared to those between 32 and 36.6 wks. At T30, AI and OFA remained higher in BM < 32 wks, while HH, HPI, MUFA/SFA and DFAs ratio were lower than in BM > 37 wks (Table 3).

## 4. Discussion

### 4.1. Composition of Breast Milk Across Different Gestational Ages

This study provides novel insights into the FA composition of human milk across different gestational ages, focusing on mothers of preterm, late preterm, and full-term infants. In agreement with previous reports, our results indicate that the concentration of saturated and monounsaturated fatty acids differs according to gestational age and during the transition phase of lactation (T7); however, these differences decrease as lactation progresses [24,25,26]. Human milk composition can improve newborns’ outcomes, whereby it is important to take into account the factors that can influence BM [27]. Zhao et al. reported that full-term BM showed greater concentration of AA and DHA compared to preterm BM, and even if that lipidome profile changed across different lactation stages in different gestational ages, no statistical variations were reported in mature human milk among different gestational ages. However, they did not report data about mother nutrition and supplementation during pregnancy, but this is a factor that can influence BM composition [27]. Most of the differences in the lipidic profile between preterm and full-term milk were made up of fatty acid ester of hydroxyl fatty acid (FAHFA) and diacylglycerol (DAG). FAHFA and DAG level concentrations were higher in human milk of different GAs compared to term human milk. It is possible that the breast tissue of premature mothers, which presented a higher proportion of adipose tissue, also caused a high secretion of FAHFA as a compensatory effect to satisfy the greater nutritional requirements of premature infants. FAHFA can be influenced by a PUFA-rich diet and dietary sources, and can promote metabolism and gut maturation of preterm newborns, while DAG could enhance the immune function of preterm newborns [28].

### 4.2. Characteristics of Transitional and Mature Milk

Thakkar et al. reported that preterm transitional milk did not show a difference between preterm (<32.6 wks) and term group (>37 wks). In contrast, preterm mature milk contained significantly higher lauric acid, myristic acid, linoleic acid, sum SFA, and lower levels of palmitoleic acid and oleic acid compared to the term group. In our analysis, preterm infants were divided into two categories: <32 wks and between 32 and 36.6 wks. A statistically significant difference was revealed between the transitional milk of preterm and term newborns. Term transitional milk showed a lower level of dihomo-gamma-linolenic acid and AA compared to preterm newborns between 32 and 36.6 wks. Instead, preterm mature milk had a greater level of SFA and myristic acid but a lower level of MUFA and MUFA/SFA compared to term mature milk [29]. Myristic acid, beyond serving as an energy substrate, is involved in post-translational modifications, such as protein N-myristoylation, essential for cell signalling and metabolic regulation. It has also been shown to modulate microbial populations and to inhibit certain bacterial ATP-binding cassette (ABC) transporters, thus potentially contributing to host–microbiota homeostasis in early life [30,31]. Furthermore, the MUFA/SFA ratio suggests altered enzymatic activity or hormonal regulation in mothers of preterm infants [32]. Importantly, erucic and nervonic acids—key components for neuronal membrane and myelin biosynthesis—were significantly lower in preterm transitional milk, potentially impacting neurodevelopmental outcomes in this vulnerable population [33]. At T7, the PUFA/SFA ratio was significantly lower in preterm <32 wks compared to those between 32 and 36.6 wks, indicating a higher SFA level in preterm transitional milk. This could reflect an adaptive mechanism aimed at improving digestibility and energy availability in preterm infants, whose gastrointestinal and pancreatic enzyme systems, such as lipase and amylase, are immature until after 6 months of age and less efficient at processing complex lipids. Indeed, medium-chain and saturated fatty acids are more readily hydrolysed and absorbed in the neonatal gut without the need for bile salts or carnitine-mediated mitochondrial entry, thereby providing a metabolically efficient energy source during early life [34]. Since neonates, particularly preterm infants, have limited capacity for endogenous elongation and desaturation of fatty acids, the dietary provision of long-chain MUFAs through breast milk is critical in the early postnatal period [35]. In addition, higher levels of long-chain PUFAs were observed in preterm transitional milk of the 32–36.6-wk group compared to the full-term group. These FAs were involved in eicosanoid synthesis and immune regulation [36]. This finding diverges from some previous reports suggesting lower PUFA content in preterm milk and may point to the existence of distinct regulatory mechanisms governing PUFA synthesis and secretion in the mammary gland [7,37].

### 4.3. Diet and Mother Supplementation During Pregnancy

One possible explanation is that PUFA levels are more strongly influenced by maternal characteristics, such as pre-pregnancy BMI, age, or dietary intake, rather than gestational age and sex [37]. For instance, maternal PUFA status is known to be diet-dependent, particularly with respect to the intake of essential fatty acids, such as linoleic and α-linolenic acids and their long-chain derivatives, such as DHA and arachidonic acid [38]. The nutrition and supplementation profile of our mothers was similar, and weekly consumption of fish was positively related to DHA, EPA and some omega-3. This shows that awareness of the importance of eating correctly and taking 100–200 mg/day DHA-EPA supplements is spreading among mothers, which positively impacts the health of newborns. Weekly consumption of eggs was not correlated with fatty acids, as also reported in a systematic review conducted by Bravi et al. [17]. Although seafood is the best source of DHA and EPA, it is not recommended to consume more than two servings of fish per week because fish can contain dioxins, organic mercury, and other toxic elements that are harmful to the health of the mother and child [14]. Furthermore, a diet rich in dairy products appears to increase palmitic acid concentration in BM. However, other studies have demonstrated that a high-fat or low-fat diet, a vegetarian or non-vegetarian diet, or an organic or non-organic diet does not alter palmitic acid concentrations [17]. In our study, we did not find an influence of diet on palmitic acid levels. Palmitic acid, which represents 20–30% of the total fat in BM, can also be found in meat and dairy products, accounting for 50–60% of total fat [39]. Furthermore, it was reported that a high-fat diet increases stearic acid concentration in BM; however, in our study, weekly cheese consumption was not correlated to it [17]. Floris et al. conducted a pooled data analysis, which demonstrated that the lipid profile of DHA in BM was similar between different gestational ages; probably these FAs were greatly influenced by the maternal dietary influence and by specific regional differences in which fish and marine products are consumed more [21,40]. The feeding habits of the mothers as well as their geographical origin were comparable, and this could explain the overlapping of the lipid profiles of the BM analysed. Dietary supplementation with PUFA may play a role in reducing the incidence of inflammatory bowel disease and necrotising enterocolitis, also enriching the intestinal microbiota [41]. However, breastfeeding remains the gold standard for the development of a balanced gut microbiota, and this may also reduce the incidence of necrotising enterocolitis [42,43,44].

### 4.4. Lipid Quality Indices

The lipid quality of a particular food product can be assessed not only by quantifying individual fatty acids, but also by specific ratios and nutritional indices commonly used to evaluate the potential health impact of the fat composition. AI reflects the atherogenic potential of fatty acids and is the ratio between SFA, which are considered pro-atherogenic, and UFA, which are considered anti-atherogenic. Pietrzak-Fiećko et al. and Purkiewicz A. et al. reported mean AI values of 1.12 ± 0.43 and 1.47, mean IT values of 0.84 ± 0.23 and 1.60, and HH ratios of 1.67 ± 0.65 and 1.21, respectively, without specifying the gestational age of the BM analysed [2,20]. In our study, mature milk <32 wks showed a median AI of 1.404, significantly higher than that of term milk. As for IT, our data showed median values ranging from 1.128 to 1.168, with no statistically significant differences among. Regarding the HH ratio, which reflects the balance between hypocholesterolemic FA and hypercholesterolemic FA, our results showed higher values of HH in term milk, with a median of 1.262, compared to preterm milk <32 wks, suggesting a more favourable lipid profile in term BM. DFA is the sum of MUFA, PUFA, and stearic acid. DFA values reported in the literature range between 51.68 and 70.88%, with such variability largely influenced by the maternal diet, particularly the intake of foods rich in specific fatty acids, such as LA, ALA, DHA and AA. Our mature preterm milk <32 wks exhibited a lower median of 55.54% for DFA and a higher median of 38.95% for OFA compared to mature term milk. OFA, defined as the sum of lauric, myristic, and palmitic acids, estimates the potentially atherogenic effect. Purkiewicz A et al. reported a mean of 41.10% for OFA consistent with values found in the literature. Although potentially atherogenic, lauric and myristic acids also exhibit notable antimicrobial and antiviral properties, which may play a role in protecting the newborn [20].

### 4.5. Implications of the Study

The results of this study contribute to expanding knowledge on the lipid composition of BM at different gestational ages, promoting appropriate nutrition and supplementation during pregnancy. Our study also aims to raise awareness about the benefits of breastfeeding, which remains the optimal nutritional choice for infants. When breastfeeding is not possible, the use of donor milk should be considered in specific cases, such as very premature and very low birth weight infants [45]. Finally, the use of enriched formula milk whose nutritional properties closely resemble those of BM should not be excluded. The science of human milk opens new therapeutic avenues for minimising the health consequences in preterm infants, who are particularly vulnerable to nutrient deprivation, by addressing the nutritional gaps that arise shortly after birth.

### 4.6. Limitations of the Study

This study has several limitations. First, the case report form used to collect dietary information has not yet been validated. Second, colostrum samples were not included in the analysis, potentially missing important early nutritional data. Additionally, DHA levels were not assessed in the infants’ blood, limiting our ability to correlate BM composition with neonatal DHA status. Finally, we analysed only maternal BM and did not include donor or fortified milk, which may have different nutritional profiles and clinical implications.

## 5. Conclusions

Interpretations of these findings must consider the infant’s physiological needs across different gestational stages. While the total lipid quantity of preterm milk is not altered, the relative enrichment in saturated and medium-chain fatty acids may offer a metabolic advantage by facilitating rapid energy mobilisation. Conversely, the diminished presence of MUFAs and long-chain unsaturated fatty acids in preterm milk may give rise to concerns regarding the provision of adequate support for neurodevelopment and membrane biosynthesis, particularly within the context of the underdeveloped central nervous system. Our data demonstrate that the total fatty acid content remains constant across gestational ages, while the profile of specific lipid subclasses is significantly modulated. These compositional adaptations are likely the result of evolutionary strategies aimed at optimising neonatal survival and development according to gestational maturity. Further research is necessary to evaluate the long-term functional implications of these variations, particularly within the framework of neurodevelopmental trajectories and the maturation of gut microbiota. Therefore, the absence of GA dependent variation in milk PUFA levels in our study should not be interpreted as a lack of biological relevance. Rather, it should be considered a reflection of complex and possibly individualised maternal–lactational regulatory networks. It is imperative to comprehend these mechanisms to develop targeted nutritional interventions in neonatal care, particularly for infants born prematurely.

## Figures and Tables

**Table 1:** Baseline characteristics of mothers.

	<32 wks	32–36.6 wks	>37 wks	*p*-Value
	Median (Q1–Q3)			
Number	25	39	34	
Mother age (years)	33 (27–37)	34 (30–37)	32 (30–37)	0.624
BMI	22.7 (20.7–30)	24.6 (22.5–27)	23 (21.8–25.2)	0.667
Number of Pregnancies	1 (0–1)	1 (1–2)	1 (0–2)	0.413
Fish consumption/week	2.0 (1–2)	2.0 (1–2)	2 (1–2)	0.763
Red meat consumption/week	2.0 (1–2)	1.8 (1–2)	1 (1–2)	0.449
White consumption/week	3.0 (2–3)	2.0 (2–3)	2 (2–3)	0.152
Eggs consumption/week	1.3 (1–2)	2.0 (1–2)	2 (1–2)	0.501
Cheese consumption/week	3.0 (1–5)	2.0 (1–3.5)	2 (1–4)	0.519
Dairy products consumption/week	2.0 (1–3)	1.0 (1–2.3)	1.5 (1–2)	0.513
Dried fruit consumption/week	1.0 (0–2)	1.0 (0–4)	2.8 (0–7)	0.289
Vegetables consumption/week	7.0 (4–7)	7.0 (6–7)	7.0 (6–7)	0.289
DHA assumption (%)	62	63	82	0.185

Q1: first quartile; Q3: third Quartile; BMI: body mass index.

**Table 2:** Fatty acid profiles of mothers’ breast milk (g/100 g of total FA) at T7 and T30.

		T7			T30		
Fatty Acids	Chain Length	<32 wks	32–36.6 wks	≥37 wks	<32 wks	32–36.6 wks	≥37 wks
**SFA**		51.371 (48.348–54.041) ^a^	48.18 (44.643–51.118) ^a^	49.596 (47.767–51.174)	51.647 (49.05–54.919) ^c^	48.469 (47.082–50.795)	48.751 (44.803–52.327) ^c^
**Caproic acid (C6:0)**	SCFA	0.1 (0.1–0.1)	0.1 (0.1–0.2)	0.1 (0.1–0.2)	0.1 (0.1–0.2)	0.1 (0.1–0.15)	0.1 (0.1–0.2)
**Caprylic acid (C8:0)**	MCFA	0.2 (0.1–0.2)	0.2 (0.1–0.3)	0.2 (0.2–0.2)	0.2 (0.2–0.3)	0.2 (0.2–0.3)	0.2 (0.2–0.3)
**Capric acid (C10:0)**	MCFA	1.1 (0.8–1.5)	0.95 (0.4–1.4)	1.2 (0.8–1.4)	1.1 (0.9–1.6)	1.55 (1.05–1.8)	1.2 (0.9–1.5)
**Undecylic acid (C11:0)**	MCFA	0.1 (0.1–0.1)	0.1 (0.1–0.2)	0.1 (0.1–0.2)	0.1 (0.1–0.2)	0.1 (0.1–0.1)	0.1 (0.1–0.2)
**Lauric acid (C12:0)**	MCFA	6.5 (5.4–7.9)	5.75 (3.5–7.8)	5.8 (4.8–7.2)	7 (5.6–8.6)	6.8 (5.35–8.3)	5.8 (4.8–7.2)
**Tridecylic acid (C13:0)**	LCFA	0.1 (0.1–0.2)	0.2 (0.1–0.2)	0.1 (0.1–0.2)	0.1 (0.1–0.2)	0.1 (0.1–0.15)	0.2 (0.1–0.2)
**Myristic acid (C14:0)**	LCFA	8.4 (6.5–9.2) ^a^	6.6 (5.2–8.8) ^a^	6.7 (5.5–8.2)	8.2 (6–9) ^c^	7.15 (6.15–8.8)	7.1 (5.4–7.5) ^c^
**Pentadecylic acid (C15:0)**	LCFA	0.5 (0.4–0.5)	0.5 (0.4–0.6)	0.4 (0.4–0.5)	0.5 (0.4–0.6)	0.45 (0.4–0.5)	0.5 (0.4–0.6)
**Palmitic acid (C16:0)**	LCFA	24.2 (23.3–25.6)	22.5 (21–24.3)	24.5 (22.2–25.5)	22.9 (21.6–25.2)	21.7 (20.55–22.9)	22.9 (21–23.6)
**Margaric acid (C17:0)**	LCFA	0.5 (0.5–0.6)	0.5 (0.5–0.6)	0.5 (0.5–0.6)	0.6 (0.5–0.7)	0.5 (0.45–0.6)	0.5 (0.5–0.7)
**Stearic acid (C18:0)**	LCFA	7 (6.2–7.9)	5.85 (5.1–7.1)	6.8 (6.1–7.6)	7.5 (5.7–9.1)	7 (6.45–7.3)	7.3 (5.5–8.5)
**Arachidic acid (C20:0)**	VLCFA	0.6 (0.5–0.7)	0.7 (0.5–1)	0.7 (0.5–0.9)	0.8 (0.6–1.1)	0.6 (0.5–0.8)	0.7 (0.6–1)
**Heneicosylic acid (C21:0)**	VLCFA	0.3 (0.2–0.4)	0.4 (0.3–0.7)	0.4 (0.3–0.5)	0.4 (0.3–0.6)	0.3 (0.2–0.45)	0.4 (0.3–0.6)
**Behenic acid (C22:0)**	VLCFA	0.5 (0.4–0.6)	0.6 (0.4–0.9)	0.6 (0.4–0.7)	0.6 (0.4–0.9)	0.45 (0.3–0.6)	0.5 (0.4–0.8)
**Tricosylic acid (C23:0)**	VLCFA	0.4 (0.3–0.4)	0.5 (0.3–0.7)	0.4 (0.3–0.6)	0.4 (0.3–0.7)	0.35 (0.2–0.5)	0.4 (0.3–0.7)
**Lignoceric acid (C24:0)**	VLCFA	0.7 (0.5–0.9)	0.9 (0.6–1.3)	0.8 (0.6–1)	0.8 (0.5–1.3)	0.65 (0.4–0.85)	0.8 (0.5–1.2)
**MUFA**		33.631 (32.489–36.498) ^a^	36.585 (33.679–38.899) ^a^	35.946 (34.542–37.2)	33.501 (30.92–35.584) ^c^	35.133 (33.518–36.941)	36.23 (34.492–38.621) ^c^
**Myristoleic acid (C14:1, n-5)**	LCFA	0.3 (0.3–0.3)	0.3 (0.3–0.5)	0.3 (0.3–0.3)	0.3 (0.3–0.4)	0.3 (0.25–0.4)	0.3 (0.3–0.5)
**Pentadecenoic acid (C15:1, n-5)**	LCFA	0.2 (0.1–0.2)	0.2 (0.1–0.3)	0.2 (0.1–0.2)	0.2 (0.1–0.3)	0.2 (0.1–0.2)	0.2 (0.1–0.3)
**Palmitoleic acid (C16:1, n-7)**	LCFA	1.5 (1.3–2.3)	1.65 (1.4–1.9)	1.5 (1.3–1.8)	1.7 (1.1–1.8)	1.5 (1.35–1.95)	1.5 (1.3–2)
**Heptadecenoic acid (C17:1, n-7)**	LCFA	0.4 (0.3–0.4)	0.4 (0.3–0.6)	0.4 (0.3–0.5)	0.4 (0.3–0.6)	0.4 (0.3–0.45)	0.4 (0.3–0.5)
**Elaidic acid (C18:1, n-9)**	LCFA	0.2 (0.1–0.2)	0.2 (0.1–0.4)	0.2 (0.2–0.3)	0.2 (0.1–0.4)	0.2 (0.1–0.25)	0.2 (0.1–0.3)
**Oleic acid (C18:1, n-9)**	LCFA	28.8 (27.5–31.1)	31.25 (28.2–33.4)	30.8 (29.4–31.5)	29.1 (24.1–30.6)	30.65 (29.05–32.15)	30.6 (28.6–32.4)
**Gondoic acid (C20:1, n-9)**	VLCFA	1 (0.8–1.1)	1.1 (1–1.3)	1 (0.9–1.1)	0.9 (0.8–1.1)	0.8 (0.75–0.95)	0.9 (0.8–1.1)
**Erucic acid (C22:1, n-9)**	VLCFA	0.6 (0.5–0.8) ^a^	0.85 (0.6–1.1) ^a^	0.7 (0.6–1)	0.7 (0.5–1.1)	0.6 (0.5–0.8)	0.7 (0.5–1)
**Nervonic acid (C24:1, n-9)**	VLCFA	0.4 (0.3–0.6) ^a^	0.6 (0.4–0.8) ^a^	0.5 (0.4–0.6)	0.5 (0.3–0.8)	0.4 (0.3–0.5)	0.4 (0.3–0.6)
**PUFA**		14.384 (12.724–15.683)	15.571 (13.779–17.408)	13.985 (13.133–16.227)	14.861 (13.047–17.019)	15.439 (13.114–18.368)	14.1 (12.635–18.056)
**ω-6**		12.17 (10.982–13.449)	13.297 (11.51–14.2)	12.313 (10.251–13.575)	0.4 (0.3–0.5)	0.3 (0.25–0.4)	0.4 (0.3–0.5)
**Linolelaidic acid (C18:2, n-6)**	LCFA	0.3 (0.3–0.4)	0.4 (0.3–0.6)	0.4 (0.3–0.4)	0.4 (0.3–0.5)	0.3 (0.25–0.4)	0.4 (0.3–0.5)
**Linoleic acid (C18:2, n-6)**	LCFA	8.4 (6.4–9.6)	8.1 (6.4–10.1)	8.3 (6.6–9.7)	7.7 (6.7–9)	9.2 (7.15–11.45)	7.8 (6.7–11.1)
**Gamolenic acid (C18:3, n-6)**	LCFA	0.3 (0.2–0.3)	0.35 (0.3–0.5)	0.3 (0.3–0.4)	0.3 (0.3–0.5)	0.3 (0.25–0.4)	0.3 (0.3–0.6)
**Eicosadienoic acid (C20:2, n-6)**	VLCFA	1 (0.9–1.2)	1.25 (0.9–1.6)	1.1 (0.9–1.3)	1 (0.8–1.4)	0.9 (0.8–1)	1 (0.9–1.3)
**Dihomo-gamma-linolenic acid (C20:3, n-6)**	VLCFA	0.8 (0.8–1)	0.9 (0.8–1.2) ^b^	0.8 (0.7–0.8) ^b^	0.7 (0.6–1)	0.7 (0.6–1)	0.8 (0.7–1)
**Arachidonic acid (C20:4, n-6)**	VLCFA	0.9 (0.6–1.1)	0.95 (0.7–1.4) ^b^	0.8 (0.7–0.9) ^b^	0.8 (0.6–1.2)	0.85 (0.7–1.1)	0.8 (0.7–1.2)
**Docosadienoic acid (C22:2, n-6)**	VLCFA	0.5 (0.4–0.6)	0.65 (0.4–1)	0.6 (0.4–0.7)	0.6 (0.4–0.9)	0.45 (0.3–0.6)	0.5 (0.4–0.8)
**ω-3**		2.153 (1.571–2.388)	2.44 (1.834–3.518)	2.172 (1.908–2.64)	2.519 (2.045–3.263)	2.076 (1.866–2.7)	2.604 (1.863–3.827)
**Linolenic acid (C18:3, n-3)**	LCFA	0.5 (0.4–0.6)	0.65 (0.5–0.9)	0.6 (0.5–0.7)	0.7 (0.6–0.8)	0.7 (0.5–0.85)	0.8 (0.6–0.9)
**Dihomolinolenic acid (C20:3, n-3)**	VLCFA	0.3 (0.3–0.4)	0.4 (0.3–0.6)	0.4 (0.3–0.5)	0.3 (0.2–0.5)	0.3 (0.2–0.4)	0.4 (0.3–0.5)
**Eicosapentaenoic acid (C20:5, n-3)**	VLCFA	0.3 (0.2–0.4)	0.4 (0.2–0.6)	0.4 (0.3–0.5)	0.4 (0.3–0.6)	0.3 (0.2–0.4)	0.4 (0.3–0.6)
**Docosahexaenoic acid (C22:6, n-3)**	VLCFA	0.9 (0.7–1)	1.1 (0.8–1.6)	1 (0.8–1.2)	1 (0.7–1.4)	0.85 (0.7–1.1)	1 (0.7–1.5)

Data are reported as median ± interquartile range (Q1–Q3); SCFA: Short-chain fatty acid; MCFA: Medium-chain fatty acid; LCFA: Long-chain fatty acid; VLCFA: Very long-chain fatty acid; PUFA: polyunsaturated fatty acid; SFA: saturated fatty acid; MUFA: monosaturated fatty acid; Superscript letters indicate significant differences between groups: ^a^ <32 wks vs. 32–36.6 wks; ^b^ 32–36.6 wks vs. ≥37 wks; ^c^ <32 wks vs. ≥37 wks.

**Table 3:** Lipid quality indices for assessing breast milk at different gestational ages.

	T7			T30		
	<32 wks	32–36.6 wks	>37 wks	<32 wks	32–36.6 wks	>37 wks
**AI**	1.354 (1.061–1.511) ^a^	1.069 (0.863–1.296) ^a^	1.137 (1.013–1.321)	1.404 (1.082–1.486) ^b^	1.134 (1.036–1.264)	1.103 (0.893–1.255) ^b^
**IT**	1.286 (1.18–1.539) ^a^	1.141 (0.91–1.271) ^a^	1.229 (1.093–1.286)	1.248 (1.074–1.405)	1.168 (1.039–1.241)	1.128 (0.906–1.308)
**HH**	1.129 (1–1.264) ^a^	1.306 (1.172–1.59) ^a^	1.25 (1.126–1.333)	1.13 (0.956–1.309) ^b^	1.302 (1.181–1.399)	1.262 (1.158–1.571) ^b^
**UI**	57.866 (52.989–62.928) ^a^	60.41 (57.758–70.632) ^a^	60.562 (57.571–62.3)	72.056 (65.908–79.058)	75.412 (70.521–79.129)	76.377 (69.096–84.633)
**HPI**	0.739 (0.662–0.943) ^a^	0.936 (0.772–1.159) ^a^	0.88 (0.757–0.987)	0.712 (0.673–0.924) ^b^	0.885 (0.791–0.965)	0.907 (0.797–1.12) ^b^
**FLQ (%)**	1.228 (0.887–1.354)	1.485 (1.082–2.108)	1.228 (1.086–1.56)	1.46 (1.072–1.991)	1.171 (0.997–1.473)	1.447 (1.033–2.195)
**LA/ALA**	15.825 (11.563–19.359)	12.305 (7.909–18.074)	12.482 (9.534–18.355)	9.474 (7.309–12.571)	13.905 (10.481–18.882)	9.3 (7.458–13.433)
**TFA (%)**	0.477 (0.395–0.56)	0.608 (0.441–0.957)	0.521 (0.417–0.671)	0.635 (0.426–0.847)	0.525 (0.373–0.625)	0.616 (0.472–0.99)
**DFAs (%)**	55.793 (52.866–57.885) ^a^	59.04 (56.249–61.306) ^a^	57.498 (55.917–59.364)	55.54 (52.315–58.698) ^b^	58.2455 (56.439–59.986)	58.451 (56.243–60.593) ^b^
**OFA (%)**	39.144 (36.255–41.699) ^a^	35.453 (32.224–38.459) ^a^	36.498 (35.374–38.426)	38.953 (35.524–40.62) ^b^	35.9435 (34.659–38.226)	35.072 (31.743–37.794) ^b^
**PUFA/SFA**	0.289 (0.234–0.308)^a^	0.328 (0.277–0.389)^a^	0.284 (0.259–0.341)	0.28 (0.228–0.343)	0.324 (0.268–0.373)	0.3 (0.242–0.397)
**PUFA/MUFA**	0.418 (0.364–0.48)	0.415 (0.387–0.487)	0.38 (0.358–0.469)	0.443 (0.372–0.509)	0.417 (0.385–0.512)	0.371 (0.358–0.508)
**MUFA/SFA**	0.657 (0.602–0.739) ^a^	0.755 (0.655–0.892) ^a^	0.722 (0.692–0.765)	0.641 (0.55–0.758) ^b^	0.733 (0.666–0.804)	0.709 (0.657–0.856) ^b^
**ω-6/ω-3**	6.147 (4.803–7.49)	5.244 (3.375–6.748)	5.055 (4.214–6.98)	4.523 (3.258–6.014)	5.519 (4.736–7.582)	4.229 (3.751–6.077)

Data are reported as median ± interquartile range (Q1–Q3); AI: Index of atherogenicity; IT: Index of thrombogenicity; HH: Hypocholesterolemic/Hypercholesterolemic ratio; UI: Unsaturation index; HPI: Health-promoting index; FLQ: Fish lipid quality/flesh lipid quality; LA/ALA: Linoleic acid/α-linolenic acid ratio; TFA: Trans fatty acid; DFAs: Desirable fatty acids, OFAs: hypercholesterolemic fatty acids. Superscript letters indicate significant differences between groups: ^a^ <32 wks vs. 32–36.6 wks; ^b^ <32 wks vs. ≥37 wks.

## Data Availability

The raw data supporting the conclusions of this article will be made available by the authors on request.

## Supplementary Materials

The following supporting information can be downloaded at: https://www.mdpi.com/article/10.3390/nu17162672/s1, Table S1: SIM parameters for FAMEs quantification in breast milk.; Table S2: Method validation parameters for GC–MS-based quantification of FAMEs identified in the breast milk samples.; Table S3:. Method validation parameters for GC–MS-based quantification of FAMEs identified in the breast milk samples.; Table S4: Pearson’s correlation between fatty acid profile at T7 and weekly eating habits.; Table S5: Pearson’s correlation between fatty acid profile at T30 and weekly eating habits.

## Abbreviations

The following abbreviations are used in this manuscript:

BM	Breast milk
TAGs	Triglycerides
FFA	Free fatty Acid
GA	Gestational age
SFA	Saturated fatty acid
MUFA	Monounsaturated fatty acid
PUFA	Polyunsaturated fatty acid
EFA	Essential fatty acid
ALA	α-linolenic acid
LA	Linoleic acid
AA	Arachidonic acid
FA	Fatty acids
DHA	Docosahexaenoic acid
EPA	Eicosapentanoic acid
FAME	Fatty Acid Methyl Esters
SD	Standard deviation
LOQ	Limits of quantification
LOD	Limit of detection
IT	Index of thrombogenicity
HH	Hypocholesterolemic/Hypercholesterolemic ratio
UI	Unsaturation index
HPI	Health-promoting index
FLQ	Fish lipid quality/flesh lipid quality
LA/ALA	Linoleic acid/α-linolenic acid ratio
TFA	Trans fatty acid
DFAs	Desirable fatty acids
OFA	Hypercholesterolemic fatty acids
FAHFA	Fatty acid ester of hydroxyl fatty acid
DAG	Diacylglycerol
