# Supplementary material for: Fatty Acid Profiling of Breast Milk at Different Gestational Ages
Source: Nutrients. 2025 Aug 19;17(16):2672. doi: 10.3390/nu17162672 (PMC12389676; doi:10.3390/nu17162672)
Supplement: Supplementary file 1 [file nutrients-17-02672-s001.zip › nutrients-3756508-S1.pdf]

Maternal Nutrition and Health
Case Report Form During Pregnancy

SECTION 1 – PERSONAL INFORMATION

First Name	
Last Name	
Age at delivery	
Height (cm)	
Pre-pregnancy weight (kg)	
Weight gain during pregnancy (kg)	
Number of pregnancies (including current)	
Type of infant feeding in previous pregnancies (if applicable)	☐ Exclusive breastfeeding<br>☐ Mixed feeding<br>☐ Formula only

SECTION 2 – DIETARY HABITS (Average weekly intake)

Please indicate your average weekly intake during pregnancy for the following food categories

**Food item**	**Frequency (times per week)**	**Additional Information**
**Fish**		Type(s) of fish consumed:
**White meat (e.g. chicken, turkey)**		
**Red meat (e.g. beef, lamb)**		
**Eggs**		
**Milk**		Type: ☐ Whole ☐ Semi-skimmed ☐ Skimmed ☐ Plant-based (specify):
**Dairy products (e.g. yogurt)**		
**Cheese**		Type(s):
**Nuts/seeds**		Type(s):
**Vegetables**		
**Fruit**		

SECTION 3 – SUPPLEMENTATION

Are you currently taking any DHA/EPA or omega-3 supplements?

☐ Yes ☐ No

Name of supplement:

Duration of use (months):

Other supplements used during pregnancy:

☐ Folic acid

☐ Iron

☐ Multivitamin

☐ Vitamin D

☐ Other (specify):

SECTION 4 – MATERNAL HEALTH

Do you have or have you had any maternal medical conditions?

☐ Gestational diabetes

☐ Hypertension

☐ Thyroid disorders

☐ PCOS (Polycystic Ovary Syndrome)

☐ Autoimmune diseases

☐ Other (specify):

Are you taking any medications during pregnancy?

☐ Yes ☐ No

If yes, please list them:
