# Supplementary material for: Fatty Acid Profiling of Breast Milk at Different Gestational Ages
Source: Nutrients. 2025 Aug 19;17(16):2672. doi: 10.3390/nu17162672 (PMC12389676; doi:10.3390/nu17162672)
Supplement: Supplementary file 1 [file nutrients-17-02672-s001.zip › nutrients-3756508-S2.pdf]

Table S2. SIM parameters for FAMEs quantification in breast milk.

No.	FAME	Window (*min*)	Ion (m/z)	
			Quantification	Qualification
1	**C6:0 - Caproic acid**	2.30 – 4.50	74	87
2	**C8:0 - Caprylic acid**	4.50 – 6.60	74	87
3	**C10:0 - Capric acid**	6.60 – 8.30	74	87
4	**C11:0 - Undecylic acid**	8.30 – 9.30	74	87
5	**C12:0 - Lauric acid**	9.30 – 10.30	74	87
6	**C13:0 - Tridecylic acid**	10.30 – 11.30	74	87
7	**C14:0 – Myristic acid**	11.30 – 12.15	74	87
8	**C14:1 (c9) - Myristoleic acid**	12.15 – 12.75	55	74
-	**IS**		154	-
9	**C15:0 - Pentadecylic acid**	12.75 – 13.35	74	87
10	**C15:1 (c10) - (Z)-10-Pentadecenoic acid**	13.35 – 14.05	55	74
11	**C16:0 - Palmitic acid**	14.05 – 14.75	74	87
12	**C16:1 (c9) - Palmitoleic acid**	14.75 – 15.5	55	74
13	**C17:0 - Margaric acid**	15.50 – 16.35	74	87
14	**C17:1 (c10) - (Z)-10-Heptadecenoic acid**	16.35 – 17.40	55	69
15	**C18:0 - Stearic acid**	17.40 – 18.90	74	87
16	**C18:1 (t9) - Elaidic acid**		55	97
17	**C18:1 (c9) - Oleic acid**		55	97
18	**C18:2 (t9,t12) - Linolelaidic acid**	18.90 – 19.50	81	67
19	**C18:2 (c9,c12) - Linoleic acid (LA)**	19.50 – 20.30	81	67
20	**C18:3 (c6,c9,c12) - Gamolenic acid (GLA)**	20.30 – 21.20	79	67
21	**C18:3 (c9,c12,c15) - Linolenic acid (ALA)**	21.20 – 21.90	79	95
22	**C20:0 - Arachidic acid**	21.90 – 23.80	74	87
23	**C20:1 (c11) - Gondoic acid**		55	97
24	**C20:2 (c11,c14) - Eicosadienoic acid**	23.80 – 25.70	81	67
25	**C21:0 - Heneicosylic acid**		74	87
26	**C20:3 (c8,c11,c14) - Dihomo-gamma-linolenic acid (DGLA)**		79	80
27	**C20:4 (c5,c8,c11,c14) - Arachidonic acid (AA)**	25.70 – 26.90	79	80
28	**C20:3 (c11,c14,c17) - Dihomolinolenic acid**		95	108
29	**C22:0 - Behenic acid**	26.90 – 28.80	74	87
30	**C22:1 (c13) - Erucic acid**		320	55
31	**C20:5 (c5,c8,c11,c14,c17) - Timnodonic acid (EPA)**		79	91
32	**C22:2 (c13,c16) - 13,16-Docosadienoic acid**	28.80 – 29.70	81	67
33	**C23:0 - Tricosylic acid**	29.70 – 32.00	74	87
34	**C24:0 - Lignoceric acid**	32.00 – 33.70	74	87
35	**C24:1 (c15) - Nervonic acid**		348	55
36	**C22:6 (c4,c7,c10,c13,c16,c19) - Cervonic acid (DHA)**	33.70 – 36.00	79	91
