# Supplementary material for: Fatty Acid Profiling of Breast Milk at Different Gestational Ages
Source: Nutrients. 2025 Aug 19;17(16):2672. doi: 10.3390/nu17162672 (PMC12389676; doi:10.3390/nu17162672)
Supplement: Supplementary file 1 [file nutrients-17-02672-s001.zip › nutrients-3756508-S3.pdf]

Table S3: Method validation parameters for GC–MS-based quantification of FAMEs identified in the breast milk samples.

FAME	Range (μg/mL)	Calibration curve	R^2	LOD (μg/mL)	LOQ (μg/mL)	Conc. (μg/mL)	Intraday		Interday	
							Precision (RSD %)	Accuracy (%)	Precision (RSD %)	Accuracy (%)
**C6:0 - Caproic acid**	4 – 140	y = 3.3124x + 0.0906	0.9994	0.882	2.672	8	1.0	114.7	1.1	85.9
						16	0.2	103.0	2.4	100.2
						140	1.8	99.6	3.3	99.0
**C8:0 - Caprylic acid**	4 – 140	y = 2.7752x + 0.0897	0.9994	1.046	3.171	8	1.3	114.3	0.6	85.4
						16	0.0	104.9	3.2	100.7
						140	1.7	99.8	3.3	99.0
**C10:0 - Capric acid**	4 – 340	y = 3.0023x - 0.0609	0.9996	0.490	1.485	16	0.6	114.7	3.7	96.2
						68	1.8	94.1	3.4	106.6
						280	1.1	101.2	0.8	98.1
**C11:0 - Undecylic acid**	2 – 100	y = 2.5944x + 0.0785	0.9995	0.271	0.821	4	1.2	108.7	2.2	85.9
						34	1.8	101.9	3.7	105.2
						100	4.9	99.2	1.4	100.6
**C12:0 - Lauric acid**	4 – 340	y = 3.3471x + 0.061	0.9998	0.434	1.314	8	2.1	114.2	4.9	92.8
						32	0.9	102.7	0.9	102.8
						280	0.9	101.2	0.6	96.8
**C13:0 - Tridecylic acid**	2 – 100	y = 2.8184x + 0.0759	0.9996	0.236	0.715	4	1.5	114.8	5.3	89.2
						34	1.8	100.5	3.3	102.2
						100	2.9	99.9	1.3	99.8
**C14:0 – Myristic acid**	4 – 200	y = 3.2309x + 0.016	0.9995	0.424	1.285	16	1.5	114.6	2.9	106.0
						140	0.6	99.9	2.7	96.1
						200	2.7	100.4	1.1	102.3
**C14:1 (c9) - Myristoleic acid**	4 – 100	y = 11.048x + 0.1333	0.9999	0.094	0.284	8	1.3	102.4	5.2	104.0
						34	1.8	100.1	3.1	104.0
						100	2.0	99.6	1.2	100.9
**C15:0 - Pentadecylic acid**	4 – 100	y = 3.0675x + 0.1204	0.9999	0.319	0.967	34	1.5	98.6	2.8	102.7
						70	0.2	100.8	2.6	96.9
						100	2.1	99.8	0.7	101.4
**C15:1 (c10) - (Z)-10-Pentadecenoic acid**	2 – 100	y = 10.994x + 0.109	0.9991	0.267	0.809	34	1.8	102.1	3.0	105.1
						70	0.4	101.5	2.8	97.3
						100	2.1	99.1	1.1	100.9
**C16:0 - Palmitic acid**	6 – 510	y = 4.1888x + 0.0294	0.9997	0.463	1.404	48	2.2	101.0	0.2	99.4
						420	1.8	101.4	0.4	95.9
						510	1.0	99.1	1.0	103.3
**C16:1 (c9) - Palmitoleic acid**	4 – 100	y = 14.288x + 0.1561	0.9997	0.112	0.339	34	1.5	100.1	2.2	104.3
						70	0.3	101.4	2.3	96.8
						100	2.5	99.3	0.6	101.2
**C17:0 - Margaric acid**	4 – 100	y = 3.4134x + 0.1775	0.9996	0.560	1.697	34	1.3	101.5	2.6	104.7
						70	0.4	101.5	2.5	96.8
						100	3.9	99.1	0.2	101.2
**C17:1 (c10) - (Z)-10-Heptadecenoic acid**	4 – 100	y = 13.784x + 0.1921	0.9993	0.181	0.549	34	1.4	102.5	2.6	105.6
						70	0.4	101.8	2.6	97.2
						100	3.6	98.9	0.4	100.9
**C18:0 - Stearic acid**	4 – 340	y = 3.9891x + 0.067	0.9994	0.246	0.745	34	1.1	98.1	1.9	103.5
						70	0.4	96.8	2.5	99.5
						100	0.5	100.6	0.5	100.0
**C18:1 (t9) - Elaidic acid**	4 – 170	y = 16.75x + 0.1481	0.9995	0.197	0.596	68	1.4	97.4	4.1	112.0
						140	3.7	101.8	3.5	102.0
						340	0.6	99.2	0.6	103.5
**C18:1 (c9) - Oleic acid**	4 – 340	y = 17.247x + 0.1841	0.9991	0.622	1.884	70	3.3	106.5	0.8	102.1
						140	1.9	102.5	1.0	93.8
						170	0.5	98.4	0.5	105.3
**C18:2 (t9,t12) - Linolelaidic acid**	4 – 140	y = 11.113x + 0.1911	0.9996	0.215	0.651	32	1.1	103.3	1.4	112.3
						280	0.5	97.9	2.3	105.4
						340	2.5	100.3	0.4	98.5
**C18:2 (c9,c12) - Linoleic acid (LA)**	4 – 170	y = 10.118x + 0.2108	0.9999	0.174	0.528	34	0.9	102.9	0.2	108.7
						70	0.4	100.7	1.9	102.0
						140	0.5	99.9	0.5	99.5

**C18:3 (c6,c9,c12) - Gamolenic acid (GLA)**	4 – 140	y = 12.045x + 0.1972	0.9997	0.185	0.559	34	1.0	103.7	1.9	112.3
						70	0.2	98.3	1.9	105.7
						170	1.8	100.2	0.7	98.4
**C18:3 (c9,c12,c15) - Linolenic acid (ALA)**	4 – 170	y = 8.4667x + 0.2488	0.9993	0.492	1.492	34	1.1	109.3	1.9	111.5
						70	3.0	98.4	0.3	110.5
						140	0.4	100.3	0.4	97.1
**C20:0 - Arachidic acid**	8 – 200	y = 4.0086x + 0.3622	0.9998	0.730	2.212	34	0.8	100.6	1.2	103.4
						100	0.4	101.2	2.3	96.7
						170	4.1	99.4	0.1	101.4
**C20:1 (c11) - Gondoic acid**	8 – 170	y = 14.847x + 0.2915	0.9992	0.321	0.971	68	1.0	104.6	0.9	103.0
						140	3.5	97.2	1.8	103.4
						200	0.1	100.6	0.1	98.8
**C20:2 (c11,c14) - Eicosadienoic acid**	8 – 140	y = 10.948x + 0.4289	0.9988	0.378	1.145	34	3.1	114.6	2.1	112.2
						100	0.1	108.1	2.4	108.0
						170	0.4	97.4	0.4	98.2
**C21:0 - Heneicosylic acid**	4 – 170	y = 4.1004x + 0.2602	0.9992	0.881	2.669	34	0.8	106.9	1.2	110.3
						100	4.1	97.8	0.1	112.7
						170	0.4	100.2	0.4	95.9
**C20:3 (c8,c11,c14) - Dihomo-gamma-linolenic acid (DGLA)**	4 – 140	y = 12.59x + 0.2624	0.9992	0.282	0.854	34	0.9	108.0	1.1	114.4
						70	0.3	100.2	2.8	108.6
						140	2.9	99.6	0.3	97.9
**C20:4 (c5,c8,c11,c14) - Arachidonic acid (AA)**	4 – 140	y = 12.572x + 0.2877	0.9988	0.335	1.015	34	1.3	108.6	1.8	114.1
						70	0.5	101.3	0.8	111.2
						140	2.2	99.3	0.5	97.5
**C20:3 (c11,c14,c17) - Dihomolinolenic acid**	4 – 140	y = 11.813x + 0.204	0.9995	0.244	0.740	34	0.4	103.5	0.1	112.1
						70	0.0	97.4	2.9	105.1
						140	1.9	100.4	0.0	98.5
**C22:0 - Behenic acid**	8 – 280	y = 4.6913x + 0.3241	0.9991	1.652	5.006	68	0.7	100.8	1.1	114.2
						140	0.4	96.1	2.0	106.3
						280	3.0	100.9	1.1	98.3
**C22:1 (c13) - Erucic acid**	8 – 140	y = 32.639x + 0.4094	0.9991	0.112	0.341	34	0.6	103.0	0.6	107.2
						70	0.1	99.4	1.4	104.4
						140	2.8	102.5	0.5	98.9
**C20:5 (c5,c8,c11,c14,c17) - Timnodonic acid (EPA)**	4 – 140	y = 10.042x + 0.2355	0.9995	0.282	0.856	34	0.2	105.8	0.4	111.7
						70	0.1	98.8	1.8	105.2
						140	2.7	100.0	0.1	98.5
**C22:2 (c13,c16) - 13,16-Docosadienoic acid**	4 – 140	y = 11.398x + 0.3406	0.9974	0.544	1.649	34	0.5	102.4	2.8	113.5
						70	3.6	92.7	0.6	112.3
						140	2.8	99.0	1.1	97.2
**C23:0 - Tricosylic acid**	4 – 140	y = 4.8413x + 0.2787	0.9990	0.794	2.405	34	0.4	100.3	2.5	111.6
						70	3.7	92.7	0.2	111.4
						140	2.8	100.1	1.7	97.5
**C24:0 - Lignoceric acid**	8 – 280	y = 5.2176x + 0.4947	0.9993	1.295	3.924	68	0.4	98.3	3.0	114.5
						140	3.5	93.0	0.1	113.0
						280	2.7	100.0	1.0	97.1
**C24:1 (c15) - Nervonic acid**	4 – 140	y = 41.268x + 0.2571	0.9972	0.188	0.570	70	0.3	105.5	7.9	114.1
						100	2.5	95.6	4.5	111.9
						140	2.7	101.4	1.3	93.8
**C22:6 (c4,c7,c10,c13,c16,c19) - Cervonic acid (DHA)**	8 – 170	y = 9.8747x + 0.5507	0.9978	0.713	2.159	34	0.4	113.9	0.2	112.3
						100	2.1	107.9	0.4	111.6
						170	1.3	99.6	1.3	95.5
