# Supplementary material for: Fatty Acid Profiling of Breast Milk at Different Gestational Ages
Source: Nutrients. 2025 Aug 19;17(16):2672. doi: 10.3390/nu17162672 (PMC12389676; doi:10.3390/nu17162672)
Supplement: Supplementary file 1 [file nutrients-17-02672-s001.zip › nutrients-3756508-S4.pdf]

Table S4 : Pearson's correlation between fatty acid profile at T7 and weekly eating habits

Fatty acids	Fish consumption/ week	Read meat consumption/ week	White meat consumption/ week	Egg consumption/ week	Cheese consumption/ week	Dairy consumption/ week	Milk consumption/ week	Dried fruit consumption/ week	Vegetable consumption/ week
**SFA**	0.0136	0.0907	-0.0212	0.0446	0.1788	-0.0096	0.0414	-0.1249	-0.0027
**Caproic acid**	-0.0148	-0.1271	-0.1518	0.0348	-0.1363	0.0107	0.0132	-0.0391	0.0919
**Caprylic acid**	0.0259	-0.1619	-0.1352	-0.0962	-0.2400*	-0.0682	-0.0348	-0.0430	0.1322
**Capric acid**	-0.0677	-0.0016	0.2118	-0.0774	0.0299	-0.1283	-0.0840	-0.0119	0.0732
**Undecylic acid**	0.0553	-0.0872	-0.1222	-0.0023	-0.1814	-0.0430	0.0117	-0.0313	0.1332
**Lauric acid**	-0.0100	-0.0309	0.1531	-0.0948	0.0525	-0.2067	-0.0992	-0.0879	0.1140
**Tridecylic acid**	0.0911	-0.1620	-0.1803	0.0040	-0.1898	-0.0077	-0.0246	-0.0809	0.0941
**Myristic acid**	0.0533	-0.0225	0.0267	-0.0201	0.0887	-0.1369	-0.0462	-0.0561	0.0509
**Pentadecylic acid**	0.0819	0.0603	-0.1445	0.1226	-0.0240	0.1879	0.2320*	0.0525	0.1231
**Palmitic acid**	-0.0674	0.1602	-0.1027	0.2152	0.1368	0.2580*	0.1979	-0.0171	-0.0879
**Margaric acid**	0.0653	-0.0558	-0.1619	-0.0078	-0.0746	0.1085	0.1274	-0.0311	0.1018
**Stearic acid**	-0.0179	0.259*	0.2061	0.1065	0.1735	0.1614	0.1530	-0.0379	-0.0202
**Arachidic acid**	0.0381	-0.0806	-0.1622	-0.0099	-0.1355	0.0392	0.0503	-0.0274	0.1172
**Heneicosylic acid**	0.0176	-0.1090	-0.1980	-0.0150	-0.1481	0.0257	0.0072	-0.0517	0.1158
**Behenic acid**	0.0464	-0.0931	-0.1584	0.0090	-0.1619	0.0143	0.0233	-0.0455	0.1163
**Tricosylic acid**	0.0570	-0.0833	-0.1712	-0.0091	-0.1374	0.0222	0.0318	-0.0219	0.1118
**Lignoceric acid**	0.0365	-0.1100	-0.1733	-0.0174	-0.1588	0.0133	0.0215	-0.0301	0.1118
**MUFA**	0.0389	-0.1017	0.0177	0.0505	-0.1541	-0.0438	-0.0122	0.2630*	0.0867
**Myristoleic acid**	0.1252	-0.0930	-0.2067	-0.0365	-0.1029	0.1000	0.0546	-0.0306	0.0915
**Pentadecenoic acid**	0.0307	-0.1400	-0.1890	-0.0325	-0.1610	0.0522	0.0914	-0.0093	0.1364
**Palmitoleic acid**	-0.0402	-0.1377	-0.0166	-0.0710	0.0825	0.0507	-0.1036	0.0432	-0.0897
**Heptadecenoic acid**	0.0701	-0.1587	-0.1621	-0.0278	-0.1497	0.0105	0.0073	0.0061	0.0802
**Elaidic acid**	0.0485	-0.1322	-0.2149	-0.0108	-0.1393	-0.0048	0.0140	-0.0097	0.0932
**Oleic acid**	0.0214	-0.0128	0.0532	0.0614	-0.0659	-0.1014	-0.0684	0.2248	-0.0419

**Gondoic acid**	0.0238	-0.0797	-0.1576	0.0619	-0.2199	0.0185	0.0616	0.0645	0.1035
**Erucic acid**	0.0294	-0.1159	-0.1796	0.0089	-0.1548	0.0104	0.0213	-0.0059	0.1217
**Nervonic acid**	0.0343	-0.1207	-0.1675	0.0128	-0.1842	-0.0144	0.0011	0.0204	0.0968
**Total PUFA**	-0.0670	-0.0300	0.0139	-0.1274	-0.1092	0.0646	-0.0533	-0.0919	-0.0961
**Omega -3**	0.0465	-0.0595	-0.2530*	0.0009	-0.1756	0.0452	0.0601	0.0189	0.0926
**Linolelaidic acid**	0.0545	-0.1061	-0.1572	-0.0464	-0.1349	0.0735	0.0427	-0.0034	0.1283
**Linoleic acid**	-0.0679	0.0792	0.2430*	-0.2028	0.1254	0.0003	-0.1274	-0.0802	-0.2760*
**Gamolenic acid**	0.0694	-0.1573	-0.1665	-0.0037	-0.1235	0.0225	0.0110	-0.0258	0.1056
**Eicosadienoic acid**	0.0341	-0.0877	-0.1522	-0.0310	-0.1457	0.0290	0.0138	-0.0089	0.0637
**Dihomo-gamma-linolenic acid**	-0.0413	-0.2013	-0.1500	-0.0278	-0.1483	-0.0059	-0.1420	-0.0830	-0.0121
**Arachidonic acid**	0.0187	-0.2075	-0.1675	-0.0153	-0.1387	0.0339	-0.0747	-0.0093	0.0076
**Docosadienoic acid**	0.0488	-0.1192	-0.1896	-0.0256	-0.1380	0.0217	0.0157	-0.0454	0.0881
**Omega-6**	-0.1106	-0.0050	0.1581	-0.1625	-0.0390	0.0566	-0.1030	-0.1245	-0.1730
**Linolenic acid**	0.0453	-0.1089	-0.2780*	0.0176	-0.0919	-0.0335	0.1070	0.1377	0.1230
**Dihomolinolenic acid**	0.0637	-0.0884	-0.2208	-0.0442	-0.1262	0.0368	0.0369	-0.0065	0.0945
**Eicosapentaenoic acid**	0.0294	-0.1278	-0.1909	-0.0272	-0.1614	0.0118	0.0518	-0.0265	0.1221
**Docosahexaenoic acid**	0.0488	-0.1192	-0.1896	-0.0256	-0.1380	0.0217	0.0157	-0.0454	0.0881
**PUFA/SFA**	-0.0474	-0.0527	0.0046	-0.1076	-0.1549	0.0412	-0.0766	-0.0148	-0.0736
**PUFA/MUFA**	-0.0800	0.0050	0.0160	-0.1394	-0.0255	0.0813	-0.0262	-0.2043	-0.1278
**MUFA/SFA**	0.0165	-0.0975	0.0078	-0.0031	-0.1904	-0.0357	-0.0647	0.2040	0.0387
**Omega-6/Omega-3**	-0.1247	-0.0001	0.4240*	-0.0627	0.1058	0.0059	-0.1101	-0.1060	-0.1284

PUFA: polyunsaturated fatty acid; SFA: saturated fatty acid; MUFA: monosaturated fatty acid; *p<0,05
