# Supplementary material for: Fatty Acid Profiling of Breast Milk at Different Gestational Ages
Source: Nutrients. 2025 Aug 19;17(16):2672. doi: 10.3390/nu17162672 (PMC12389676; doi:10.3390/nu17162672)
Supplement: Supplementary file 1 [file nutrients-17-02672-s001.zip › nutrients-3756508-S5.pdf]

Table S5 : Pearson's correlation between fatty acid profile at T30 and weekly eating habits

Fatty acids	Fish consumption/ week	Read meat consumption/ week	White meat consumption/ week	Egg consumption/ week	Cheese consumption/ week	Dairy consumption/ week	Milk consumption/ week	Dried fruit consumption/ week	Vegetable consumption/ week
**SFA**	0.023	0.184	-0.020	-0.113	0.126	-0.062	-0.059	-0.141	-0.019
**Caproic acid**	0.294*	-0.112	-0.288*	0.109	0.157	0.038	-0.474*	-0.250	-0.005
**Caprylic acid**	0.321*	-0.227	-0.227	0.158	0.281*	-0.048	-0.388*	-0.255	-0.016
**Capric acid**	-0.098	-0.109	0.080	-0.081	-0.049	-0.048	0.368*	0.233	-0.172
**Undecylic acid**	0.257	-0.206	-0.309*	0.066	0.164	0.021	-0.499*	-0.199	-0.025
**Lauric acid**	-0.033	-0.081	-0.018	-0.227	-0.036	-0.036	0.118	0.009	-0.198
**Tridecylic acid**	0.304*	-0.146	-0.291*	0.149	0.110	-0.075	-0.406*	-0.124	0.054
**Myristic acid**	-0.062	-0.005	-0.113	-0.260	-0.046	-0.028	-0.122	-0.179	-0.117
**Pentadecylic acid**	0.239	-0.182	-0.385*	0.173	0.131	-0.021	-0.465*	-0.192	0.125
**Palmitic acid**	0.023	-0.197	-0.259	0.124	0.068	-0.100	-0.263	-0.126	0.093
**Margaric acid**	-0.179	0.315*	0.200	0.076	-0.054	-0.033	0.137	-0.045	0.178
**Stearic acid**	0.260*	-0.121	-0.214	0.159	0.155	-0.022	-0.378*	-0.256	0.102
**Arachidic acid**	-0.049	0.376*	0.315*	-0.097	0.049	0.046	0.138	-0.059	0.069
**Heneicosylic acid**	0.309*	-0.118	-0.243	0.157	0.172	0.036	-0.461*	-0.184	0.105
**Behenic acid**	0.289*	-0.159	-0.297*	0.194	0.151	0.040	-0.439*	-0.165	0.098
**Tricosylic acid**	0.3*	-0.145	-0.277*	0.154	0.159	0.024	-0.456*	-0.167	0.101
**Lignoceric acid**	0.288*	-0.157	-0.27*	0.158	0.126	0.006	-0.438*	-0.143	0.096
**MUFA**	0.284*	-0.158	-0.274*	0.162	0.149	0.014	-0.449*	-0.171	0.081
**Myristoleic acid**	-0.193	-0.142	0.062	0.107	-0.254	-0.045	0.237	0.259	0.026
**Pentadecenoic acid**	0.26*	-0.202	-0.306*	0.175	0.203	0.006	-0.386*	-0.108	0.128
**Palmitoleic acid**	-0.244	0.043	0.025	0.088	-0.115	-0.094	0.024	0.098	-0.063
**Heptadecenoic acid**	0.26*	-0.178	-0.262*	0.186	0.185	0.028	-0.497*	-0.209	0.081
**Elaidic acid**	0.294*	-0.137	-0.303*	0.206	0.171	0.021	-0.455*	-0.180	0.119
**Oleic acid**	-0.241	-0.054	0.131	0.047	-0.242	-0.014	0.353*	0.29*	-0.026

**Gondoic acid**	0.195	-0.113	-0.282*	0.053	0.084	-0.011	-0.438*	-0.216	-0.044
**Erucic acid**	0.277*	-0.169	-0.267*	0.179	0.145	0.020	-0.468*	-0.147	0.081
**Nervonic acid**	0.241	-0.194	-0.250	0.125	0.115	-0.026	-0.481*	-0.200	0.038
**Total PUFA**	0.181	-0.115	-0.040	0.051	0.100	0.146	-0.188	-0.088	-0.001
**Omega -3**	0.257*	-0.184	-0.212	0.112	0.095	0.089	-0.368*	-0.121	0.140
**Linolelaidic acid**	0.284*	-0.160	-0.31*	0.195	0.132	0.023	-0.445*	-0.173	0.109
**Linoleic acid**	0.019	0.026	0.165	-0.137	0.109	0.059	0.251	0.113	-0.078
**Gamolenic acid**	0.277*	-0.201	-0.281*	0.190	0.147	0.009	-0.474*	-0.196	0.113
**Eicosadienoic acid**	0.263*	-0.140	-0.223	0.117	0.160	0.002	-0.47*	-0.187	-0.002
**Dihomo-gamma-linolenic acid**	0.171	-0.325*	-0.254	0.092	0.088	-0.086	-0.541*	-0.155	-0.069
**Arachidonic acid**	0.269*	-0.243	-0.203	0.130	0.169	-0.054	-0.516*	-0.146	-0.049
**Docosadienoic acid**	0.276*	-0.151	-0.257	0.155	0.132	0.030	-0.461*	-0.186	0.073
**Omega-6**	0.098	-0.054	0.051	0.009	0.077	0.136	-0.060	-0.050	-0.067
**Linolenic acid**	0.129	-0.063	-0.276*	0.079	0.067	0.154	-0.199	0.002	0.062
**Dihomolinolenic acid**	0.284*	-0.163	-0.289*	0.162	0.139	0.004	-0.392*	-0.172	0.105
**Eicosapentaenoic acid**	0.273*	-0.156	-0.302*	0.170	0.178	0.007	-0.45*	-0.169	0.102
**Docosahexaenoic acid**	0.31*	-0.164	-0.309*	0.152	0.155	0.022	-0.442*	-0.187	0.072
**PUFA/SFA**	0.115	-0.133	-0.040	0.085	0.038	0.110	-0.102	-0.015	0.013
**PUFA/MUFA**	0.283*	-0.046	-0.081	0.017	0.217	0.154	-0.299*	-0.195	0.009
**MUFA/SFA**	-0.131	-0.152	0.028	0.119	-0.215	-0.010	0.167	0.227	0.037
**Omega-6/Omega-3**	-0.076	0.126	0.246	-0.135	0.001	-0.054	0.232	-0.030	-0.228

PUFA: polyunsaturated fatty acid; SFA: saturated fatty acid; MUFA: monosaturated fatty acid; *p<0,05
